# The Molecular Mechanisms of Glucocorticoids-Mediated Neutrophil Survival

**DOI:** 10.2174/138945011794751555

**Published:** 2011-04

**Authors:** Arash S Saffar, Heather Ashdown, Abdelilah S Gounni

**Affiliations:** Department of Immunology, Faculty of Medicine, University of Manitoba, Winnipeg, Canada

**Keywords:** Apoptosis, glucocorticoid, inflammation, neutrophils.

## Abstract

Neutrophil-dominated inflammation plays an important role in many airway diseases including asthma, chronic obstructive pulmonary disease (COPD), bronchiolitis and cystic fibrosis. In cases of asthma where neutrophil-dominated inflammation is a major contributing factor to the disease, treatment with corticosteroids can be problematic as corticosteroids have been shown to promote neutrophil survival which, in turn, accentuates neutrophilic inflammation. In light of such cases, novel targeted medications must be developed that could control neutrophilic inflammation while still maintaining their antibacterial/anti-fungal properties, thus allowing individuals to maintain effective innate immune responses to invading pathogens. The aim of this review is to describe the molecular mechanisms of neutrophil apoptosis and how these pathways are modulated by glucocorticoids. These new findings are of potential clinical value and provide further insight into treatment of neutrophilic inflammation in lung disease.

## INTRODUCTION

Neutrophils are the primary line of defense for the innate immune system. These polymorphonuclear leukocytes are produced in the bone marrow from myeloid stem cells and constitute 50-70% of white blood cells. Neutrophils in human blood are partitioned between two systems: a) the circulating pool and b) the “marginating” pool which is transiently arrested in narrow capillaries primarily in the lung [1]. Neutrophils are equally distributed and constantly exchanged between the two systems [2]. When inflammation occurs, an active process of adhesion is initiated where neutrophils are arrested from circulating in the blood vessels and are guided by graded concentrations of multiple chemo-attractants across the endothelial layer, pericyte sheath and basement membrane and into extravascular tissue [3]. Once removed from the circulating pool, neutrophils continue to migrate until they reach the site of inflammation. 

Neutrophils act by recognizing pathogen associated molecular patterns (PAMPs) through their germline encoded toll like receptors (TLRs) which help them to combat bacterial and fungal infections through phagocytosis and the release of granule contents [4]. They also express receptors for antibody (fraction crystallizable receptors: FcRs) and complement (CRs), and thus act in concert with many components of the immune system to fight infection. Bacterial killing is usually done through phagocytosis, or the uptake of individual pathogens. This process involves FcγRII and CR3 receptors that work in cooperation with their co-receptors, FcγRIIIB and CR1 [5]. Once encapsulated into the phagosome, molecules such as neutrophilic proteases and reactive oxygen species are used to digest the contents of the vacuole. Another mechanism of bacterial killing utilized by neutrophils is degranulation, which occurs when neutrophils release their pre-stored granule contents to the outside environment. Different types of granules contain various substances designed to kill target cells [5]. 

Neutrophils also have the capacity to generate massive amounts of oxygen and nitrogen intermediates through “respiratory burst” and other mechanisms. These intermediates are produced as a result of the assembly and activation of NAPDH oxidase which reduces oxygen to superoxide anion. This results in the production of intermediates containing free radicals that can be used to destroy phagocytosed microbes [6]. 

Finally, a novel anti-microbial mechanism of neutrophils has recently been characterized [7]. Neutrophil extracellular traps (NETs) are extracellular proteins mainly composed of chromatin, with specific granular components attached to them [8]. These threads form cables and three-dimensional “web-like” structures, which are “spat out” in response to cytokine-, FcR- or TLR-mediated activation [9, 10]. NETs have been shown to trap and kill both bacteria and fungi [7]. It should be noted that NET formation is also a form of cell death that does not implicate caspases or DNA fragmentation as in apoptosis [10]. 

All of the above mentioned neutrophilic activities are modulated by specific regulatory molecules. Cytokines such as tumor necrosis factor-α (TNF- α), interleukin (IL)-1β, granulocyte-colony stimulating factor (G-CSF) and granulocyte-macrophage colony stimulating factor (GM-CSF) and chemokines such as IL-8 have profound effects on neutrophils. They amplify several responses such as adhesion and respiratory burst [5]. It has now been accepted that neutrophils are not only the target of, but are also a source of various cytokines and chemokines [11]. Although neutrophils were long considered to be devoid of transcriptional activity, new evidence suggests that neutrophils constitutively or inducibly synthesize and release these mediators [12]. 

Lastly, it is important that neutrophils have the ability to discriminate between pathogenic and harmless antigens [13]. Antibodies, complement molecules and cytokines all affect neutrophil activity by specifically assisting neutrophils in distinguishing the “self” from the “non-self” and determining the course and intensity of the general immune response. Considering the aggressive nature of neutrophils, a non-specific response can cause damage to healthy tissue. When regulatory processes that control recruitment, activation and apoptosis are impaired, neutrophils may become the predominant contributor to tissue injury [5].

## NEUTROPHILS AND AIRWAY INFLAMMATORY DISEASES

Inflammation, and chronic persistence of granulocytes in tissue, is now recognized as a central process in the pathogenesis of diseases such as COPD and bronchial asthma [14]. In inflammation, the potential for neutrophils to cause tissue damage via the release of toxic reactive oxygen species and granule enzymes such as proteases is very high. For example, it has been reported in the airways that secondary necrosis of apoptotic neutrophils leads to release of cytotoxic granules, causing harm to resident structural cells [15].

Although glucocorticoids are generally considered to be the treatment of choice in many inflammatory diseases, glucocorticoid resistance in diseases such as asthma [16, 17], COPD [18], septic shock [19], idiopathic pulmonary fibrosis [20] and others has been associated with neutrophilic inflammation. It is known that neutrophilic asthma represents a fairly large proportion of the disease overall, up to 50% by some reports [21]. Although glucocorticoids lead to marked reduction of eosinophils, mast cells, T lymphocytes and macrophages in sputum, bronchoalveolar lavage and bronchial wall [21], changes in the neutrophilic components of asthma are often the opposite, with reports of increase in neutrophils after glucocorticoid therapy [22, 23]. 

The functional longevity of neutrophils at inflamed sites is generally controlled by apoptosis [14]. In treatment of asthma, clinical improvement is associated with granulocyte apoptosis and appearance of apoptotic bodies within airway macrophages [24]. As such, understanding the processes that regulate constitutive and delayed neutrophil apoptosis may assist in identifying new therapeutic targets [25].

## NEUTROPHIL APOPTOSIS

In the immune system, a fine balance is constantly maintained between apoptosis and proliferation of immune cells. Apoptosis plays an important role by preventing over-activation of immunity, consequently avoiding self-inflicted pathology. Once neutrophil apoptosis has occurred, the apoptotic cells are recognized and phagocytosed by macrophages. This prevents the release of potentially harmful neutrophilic substances into the extracellular environment. Furthermore, neutrophil elimination by macrophages is also associated with a subsequent release of anti-inflammatory molecules such as IL-10 and transforming growth factor-β [26]. During this process, the potential for neutrophils to cause tissue damage via the release of toxic reactive oxygen species and granule enzymes such as proteases is lowered by apoptosis and inflammation is discouraged. 

The above mentioned phenomena explain the association between clinical improvement in the treatment of asthma and granulocyte apoptosis. In other words, modulators of neutrophil apoptosis as a way to decrease inflammation could prove useful in the treatment of inflammatory lung disease. On the other hand, an excess of apoptosis in the immune system is also unfavorable as it predisposes the host to infections by destroying cells that are reactive to pathogens, thus providing an escape mechanism for invaders [27]. 

In the human body neutrophil apoptosis is regulated by multiple proteins. The most notable are caspases, the Bcl-2 family of proteins including myeloid cell leukemia-1 (Mcl-1), inhibitor of apoptosis proteins (IAPs), phoshatidylinositol 3- kinases (PI3Ks) and mitogen activated protein kinases (MAPKs). Any agent (e.g. glucocorticoids) capable of influencing the activity of these proteins may exert an effect on neutrophil apoptosis. 

Caspases are cysteine proteases that carry out the final stages of apoptosis by cleaving more than 200 substrate proteins at specific consensus sites [28]. Each caspase is present constitutively as a zymogen that must be proteolytically cleaved in order to be activated. Caspases can be activated through either the extrinsic or intrinsic pathway. The binding of death ligands to their receptors on the cell surface activates the extrinsic pathway (for example Fas ligand binding the Fas receptor) [29]. In contrast, the intrinsic pathway is triggered in response to death stimuli from within the cell such as DNA damage or oncogene activation [30]. The intrinsic pathway is mediated by the mitochondrion which releases initiator proteins, namely cytochrome c and second mitochondria-derived activator of caspases (Smac) aka direct IAP binding protein with low pI (DIABLO). Due to the fact that these proteins are found in the mitochondria, the release of caspase activator proteins depends on the permeability of the mitochondrial membrane. As such, the molecules responsible for controlling mitochondrial membrane integrity constitute the major checkpoint of the intrinsic pathway. They are referred to as Bcl-2 family of proteins. 

There are around 20 members of the Bcl-2 family of proteins. Bcl-2, Bcl-xl, Bcl-w, myeloid cell leukemia-1 (Mcl-1) and A1 are all anti-apoptotic family members. The major pro-apoptotic members are the cytosolic Bcl-2-associated X protein (Bax) and the outer mitochondrial membrane bound Bcl-2 homologous antagonist-killer (Bak) [31]. Activation of Bax/Bak is assured by other pro-apoptotic Bcl-2 family members such as BH3 interacting domain death agonist (Bid). Although the specific activation mechanism is not clear, it has been proposed that some proteins interact directly with Bax/Bak to activate them [32]. In another proposed mechanism, these pro-apoptotic proteins bind to anti-apoptotic factors thereby neutralizing their effect on Bax/Bak [32]. In this way, the Bcl-2 family of proteins interacts, often blocking one another’s function, and consequently the mitochondrial membrane integrity is efficiently controlled. 

In an interesting report in 1998, Moulding *et al*. compared the expression of various Bcl-2 family members in neutrophils. They reported a lack of Bcl-2 and Bcl-xL but an abundance of Mcl-1 protein [33]. Pro-survival signals such as cytokines and growth factors have been reported to induce Mcl-1 expression, whereas its levels are down-regulated during apoptosis [33]. There have been multiple mechanisms proposed for the induction or repression of Mcl-1 at the promoter level. In GM-CSF stimulated neutrophils, the Janus kinase/signal transducers and activators of transcription (JAK/STAT) pathways as well as PI3K are important for the induction of Mcl-1 [34]. Moreover, IL-3 in murine pro-B cells brings about PU.1 mediated transcription of Mcl-1 through activation of p38 MAPK [35]. The exact mode of function of Mcl-1 is not clear but it is thought to prevent loss of mitochondrial integrity and cytochrome c release. This may occur through heterodimerization with pro-apoptotic Bcl-2 family members such as Bim, Bak or Bax [34, 36]. 

Additional checkpoints for apoptosis include IAPs and PI3K. IAPs inhibit caspase activity by directly binding to them [37]. The pro-apoptotic mitochondrial proteins Smac/ DIABLO and Omi aka high temperature requirement A2 (HtrA2) antagonize IAPs, thus allowing caspase activity to pursue [38]. Secondly, the PI3Ks constitute a unique and conserved family of intracellular lipid kinases that phosphorylate the 3’hydroxyl group of phosphatidylinositol and phosphoinositides [39]. PI3K seems to function as a signaling molecule that is ubiquitously needed for survival of neutrophils. Many anti-apoptotic agents require the PI3K signal [4, 40-43]. 

One last apoptotic checkpoint to be mentioned is the MAPKs. MAPKs are a family of conserved protein kinases that phosphorylate target protein substrates and regulate a number of cellular activities including gene expression, mitosis, cell movement, metabolism, cell survival and apoptosis. Conventional MAPKs consist of three family members: the extracellular signal-regulated kinase (ERK); the c-Jun NH2-terminal kinase (JNK); and the p38 MAPK. All three family members have distinct regulation and functions [44]. 

There is evidence that ERK, p38 and JNK MAPKs are all important in regulating granulocyte apoptosis [45]. More specifically, it has been shown that p38 MAPK is the only one of the three MAPK family members that is essential for dexamethasone induced survival of human neutrophils [46, 47]. Another study done by Alvarado-Kristensson *et al*. confirmed the anti-apoptotic role of p38 MAPK by demonstrating that it can phosphorylate and inactivate caspase-3 and caspase-8 [48]. Despite these findings, many other studies have demonstrated the pro-apoptotic effects of p38 MAPK. p38 MAPK has recently been shown to be essential in inducing apoptosis in human neutrophils exposed to *Mycobacterium tuberculosis* [49]. Taken together, these findings highlight the complexity of the regulatory processes that govern the activity of MAPKs. In fact, both p38 and JNK MAPK have dual pro-/anti-apoptotic roles, while ERK appears to be primarily important in the propagation of anti-apoptotic signals [46, 47]. In the context of Dex induced survival of neutrophils, the exact role of p38 MAPK remains unknown. 

## GLUCOCORTICOIDS AND NEUTROPHIL APOPTOSIS

Glucocorticoids are part of the steroid family of hormones. They mediate their effect on target cells by directly binding to cytosolic glucocorticoid receptors (GRs). The unligated GR is normally found in the cytoplasm in a complex with multiple other proteins. Binding of the ligand then induces release of the receptor from its protein complex, dimerization and translocation to the nucleus where the GR can regulate the expression of genes. The nuclear GR binds to specific sequences of nucleic acids called Glucocorticoid response elements (GRE) in the promoter region of responsive genes and can either induce or repress transcription of various molecules including inflammatory mediators [50]. Indirect transcriptional effects of glucocorticoids result from their interaction with other transcription factors such as activating protein-1 (AP-1) and nuclear factor-κB (NF-κB) [51]. Glucocorticoids can also have non-transcriptional effects on cell activity by modulating various intra-cellular signaling pathways [52]. 

Perhaps the most important pharmacologic property of glucocorticoids is their immunosuppressive effect. Glucocorticoids have been shown to decrease expression of proinflammatory molecules and increase expression of anti-inflammatory molecules [53]. They also induce apoptosis of thymocytes, T cells and eosinophils [54]. However, although glucocorticoids are most notorious for their apoptosis inducing properties, it has become increasingly clear that they can also inhibit apoptosis and induce survival in many cell types  [55-70]. Various mechanisms for glucocorticoid mediated inhibition of apoptosis have been proposed that include up-regulation of anti-apoptotic Bcl-2 family members [55, 56, 70]; stabilization [62] and induction [68] of IAPs; activation of NF-κB [59, 61]; suppression of components of the extrinsic pathway of apoptosis [57, 65]; and induction of signaling molecules such as MAPK phosphatase-1 (MKP-1) and Serum and glucocorticoid activated kinase-1 (SGK-1) [63, 69] (Table **1**).

Many studies have reported the *in vitro* anti-apoptotic effect of glucocorticoids on human neutrophils [46, 47, 71-73]. It is now clear that glucocorticoids including dexamethasone (Dex) inhibit spontaneous neutrophil apoptosis in a concentration-dependent manner [71-76]; the anti-apoptotic effect of Dex is abolished by transcription/translation inhibitors [72, 75] and is mediated through the GR [72, 76, 77]; and unlike GM-CSF and lipopolysaccharide (LPS), Dex does not lead to activation of neutrophils, as measured by IL-8 and superoxide production [71].

There have been a number of mechanisms proposed so far for the above described phenomenon of glucocorticoid-mediated survival specific to neutrophils. One common theory is that the dominant negative GRβ isoform, which is expressed at a higher level than GRα in neutrophils, interferes with GRα mediated expression of pro-apoptotic genes. The GRβ isoform has no transcriptional activity, lacks the ability to bind ligands and inhibits GRα activity [78] which could explain the unique neutrophil response to Dex stimulation. However, current results argue against transrepression of pro-apoptotic genes as a mechanism for glucocorticoid mediated survival [46, 47].

In 2004, Chang *et al.* [79] found that bovine neutrophils treated with Dex demonstrated a decreased expression of Fas mRNA and protein, which correlated with decreased caspase-8 activity in these cells. Thus, the inhibition of the extrinsic pathway of apoptosis is a possible contributor to the glucocorticoid effect. In 2005, Madsen-Bouterse *et al.* [80] observed an increase of anti-apoptotic A1 and a decrease of pro-apoptotic Bak in Dex treated bovine neutrophils. These findings correlated with decreased activity of caspase-9, and were proposed as another possible mechanism of glucocorticoid-mediated survival.

Lastly, a few reports have confirmed the up regulation of Mcl-1 in neutrophils as a result of Dex stimulation [46, 47, 81]. Interestingly, up-regulation of the short pro-apoptotic splice variant of Mcl-1 has not been observed. It has also been demonstrated that Mcl-1 anti-sense oligonucleotides abolish Dex-induced Mcl-1 expression and survival in human neutrophils [81].

## MECHANISM OF GLUCOCORTICOID-MEDIATED NEUTROPHIL SURVIVAL

**Fig. (1). A schematic model of glucocorticoid induced survival of human neutrophil.**
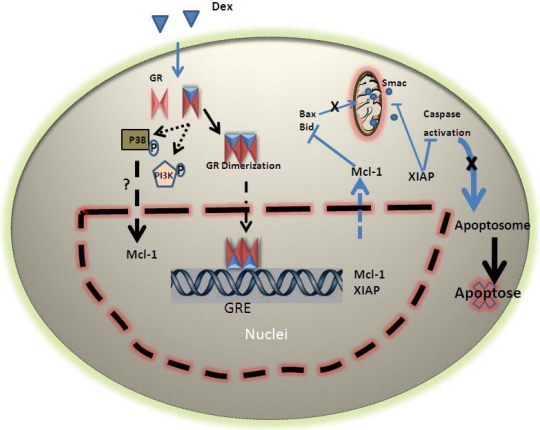
Following treatment of neutrophils with dexamethasone, the latter binds to GR inducing release of the receptor from its protein complex, dimerization and translocation to the nucleus where it can up regulate Mcl-1 and XIAP expression. The net effects are maintenance of mitochondrial integrity and suppression of caspases. GR also modulates P38 and PI3K activation that may influence Mcl-1 expression or function.

It has been suggested that glucocorticoids affect neutrophil apoptosis by suppressing various pro-apoptotic molecules. However, selective induction of the transrepressive function of GR by the GR modulator Compound A (CpdA) does not alter neutrophil apoptosis, suggesting that trans-repression is not responsible for the glucocorticoid effect [82]. Moreover, it has been observed that, in human neutrophils, the levels of pro-apoptotic proteins such as Fas, FasL, Bid and Bax are not significantly decreased following stimulation with Dex [46, 47]. Furthermore, the inhibitory impact of Dex on neutrophil apoptosis cannot be considered uniquely non-genomic, since the effect is abrogated by transcription/translation inhibitors [50, 83]. Consequently, it is now generally agreed that Dex does not act by decreasing pro-apoptotic factors [42, 81, 84, 85], but instead increases anti-apoptotic factors such as Mcl-1 and IAPs through transactivation (Fig. **1**). 

Mcl-1 is the sole anti-apoptotic member of the Bcl-2 family consistently detected in human neutrophils at the protein level [25]. It has previously been observed that glucocorticoids can inhibit apoptosis by enhancing anti-apoptotic Bcl-2 family members [55, 56, 70]. For instance, Sivertson *et al.* [81] have detected increases in Mcl-1 mRNA and protein in Dex treated human neutrophils. The consequence of the up-regulation of Mcl-1 is seen at the mitochondrial level, as Mcl-1 is an inhibitor of Bax, a pro-apoptotic Bcl-2 family member that mediates the loss of mitochondrial membrane integrity. In this way, glucocorticoids are able to regulate apoptosis at the mitochondrial level (Fig. **1**). In addition, many studies have confirmed the importance of PI3K and p38 MAPK in neutrophils and other cell types that respond to pro-survival agents by enhancing Mcl-1 [34, 35, 86]. 

Another family of proteins up-regulated as a result of glucocorticoid stimulation is the IAP family, which prevent caspase/Smac activity [37]. It has been shown that the level of XIAP, the prototypic IAP, can be maintained by glucocorticoids [46, 47, 87, 88]. This may partially account for the decreased activity of caspase-3 in neutrophil cultures [47]. 

It remains to be established whether glucocorticoids lead to direct activation of p38 MAPK and PI3K in neutrophils as it has been shown for GM-CSF [34, 47]. However, it is clear that Dex induced survival of neutrophils is suppressed by blocking PI3K and p38 but not JNK or ERK MAPK [34, 47]. It has been observed that the effect of Dex on both neutrophil survival and Mcl-1 enhancement is dependent on protein translation and signaling through PI3K and p38 MAPK [47] (Fig. **1**). These findings indicate a role for PI3K and p38 MAPK in the translation dependent enhancement of Mcl-1 by Dex; however, these signaling pathways also have alternative translation-independent anti-apoptotic effects that may in fact contribute to glucocorticoid-mediated neutrophil survival. For instance, p38 has been shown to phosphorylate and deactivate caspases-3 and 8 [48]; and PI3K-initiates pro-survival phosphorylation of Bad, Bax and caspase-9 [89, 90]. Thus, glucocorticoids may also exert non-transcriptional effects through these signaling pathways. 

## CONCLUSION

In asthma, corticosteroids have been shown to increase airway tissue neutrophils [23, 91]. They have also been shown to decrease chemotactic factors for T cells and eosinophils but not neutrophils in asthmatic airway mucosa [92]. Furthermore, glucocorticoids are reportedly inefficient in controlling increased neutrophil matrix metalloproteinase (MMP) in severe asthma. Thus, glucocorticoids alone may not be sufficient to manage certain asthma cases, particularly those characterized by neutrophil-dominated inflammation. 

From a physiological point of view, the neutrophilic reaction that follows *in vivo* glucocorticoid administration may have developed as a response to stress that boosts the innate immunity. For instance, it has been shown that although increased levels of corticosteroids in mice lead to a decrease in the lymphocyte population and suppression of the adaptive immune response, it increases neutrophil numbers and enhances anti-bacterial immunity. Mice with increased serum glucocorticoids that were exposed to *L. monocytogenes *and* S. pneumonia* demonstrated enhanced clearance of these bacterial infections [93]. 

Future studies are required in order to further clarify the mechanisms responsible for glucocorticoid induced survival of neutrophils. Determining the non-genomic effects of glucocorticoids, such as phosphorylation/dephosphorylation of key cellular proteins, or specifying the pathways downstream and upstream of PI3K/p38 MAPK may prove useful. It may also be helpful to identify the gene targets of glucocorticoids in neutrophils and to learn how induction/ repression of such genes impacts cellular function. Finally, one could even go a step further and assess the importance of the above mechanisms in survival mediated by other glucocorticoid drugs available. 

Taken together, the observations presented previously provide a model in which Dex-mediated inhibition of primary human neutrophil apoptosis is associated with increased levels of Mcl-1 and XIAP. Up-regulation of these molecules correlates with suppression of various down-stream pathways of apoptosis. These mechanisms may be initiated by GR-mediated transactivation of anti-apoptotic genes independently of, or in association with, intra-cellular signaling molecules and transcription factors. Collectively, the above results underline mechanisms through which corticosteroids undesirably modulate apoptosis of an inflammatory cell. Development of GR ligands that selectively inhibit neutrophil inflammatory function without inducing survival is thus warranted.

## Figures and Tables

**Table 1:** Currently Proposed Mechanisms for Glucocorticoid Mediated Inhibition of Apoptosis

Up-regulation of anti-apoptotic Bcl-2 family members [55, 56, 80]. Stabilization and induction of IAPs [47, 61, 62]. Activation of NF-κB [61]. Suppression of components of the extrinsic pathway of apoptosis [57, 79]. Induction of signaling molecules such as MAPK phosphatase-1 (MKP-1) and Serum and glucocorticoid activated kinase-1 (SGK-1) [63, 69].
